# Supplementary figures and images for: Cell size modulates ferroptosis susceptibility
Source: eLife. 2026 Jun 10;15:RP111544. doi: 10.7554/eLife.111544 (PMC13252968; doi:10.7554/eLife.111544)

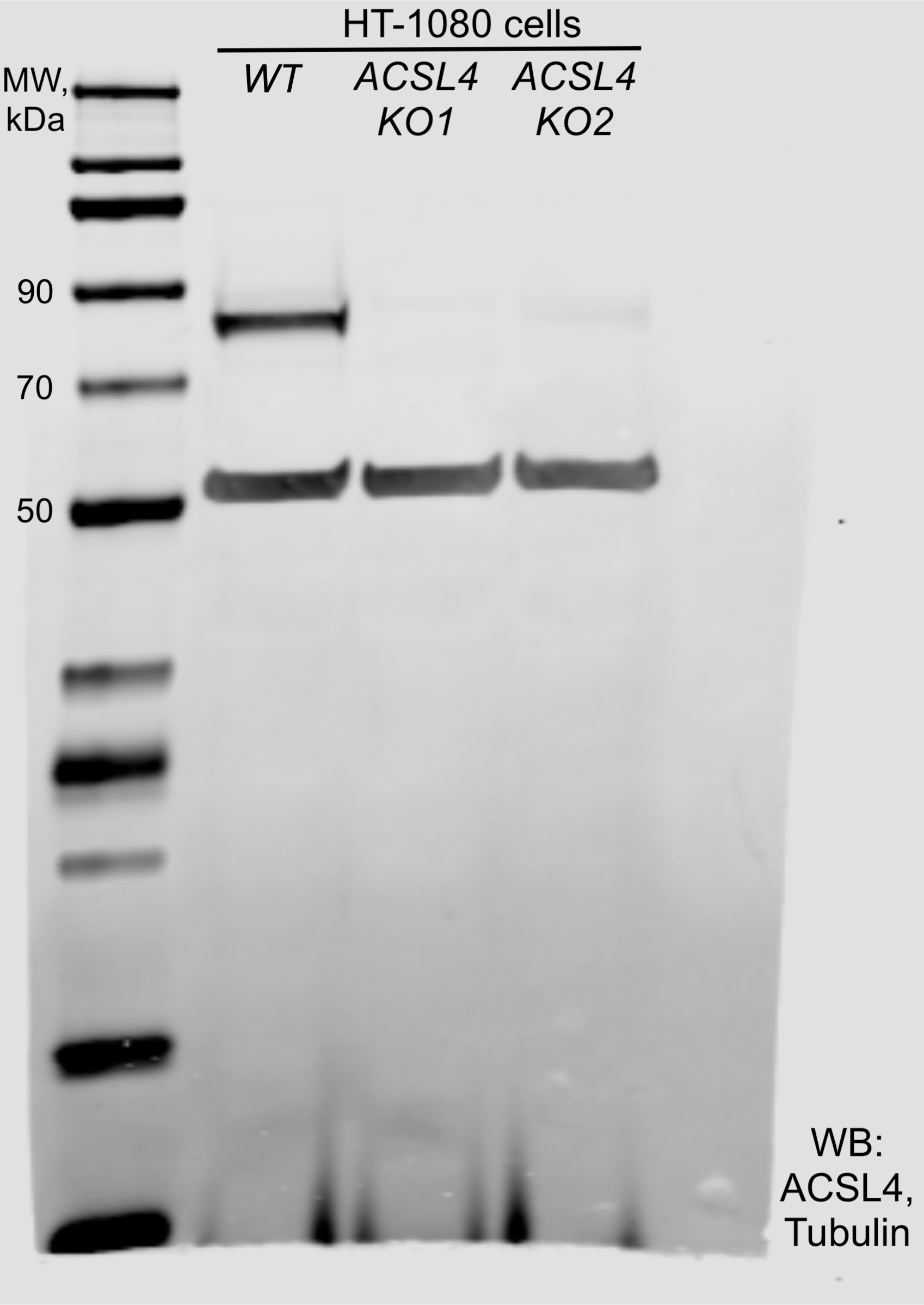

Supplement: Figure 5—source data 1. [file elife-111544-fig5-data1.zip › Figure 5_Source Data 1 - HT1080_WB_ACSL4.tif]

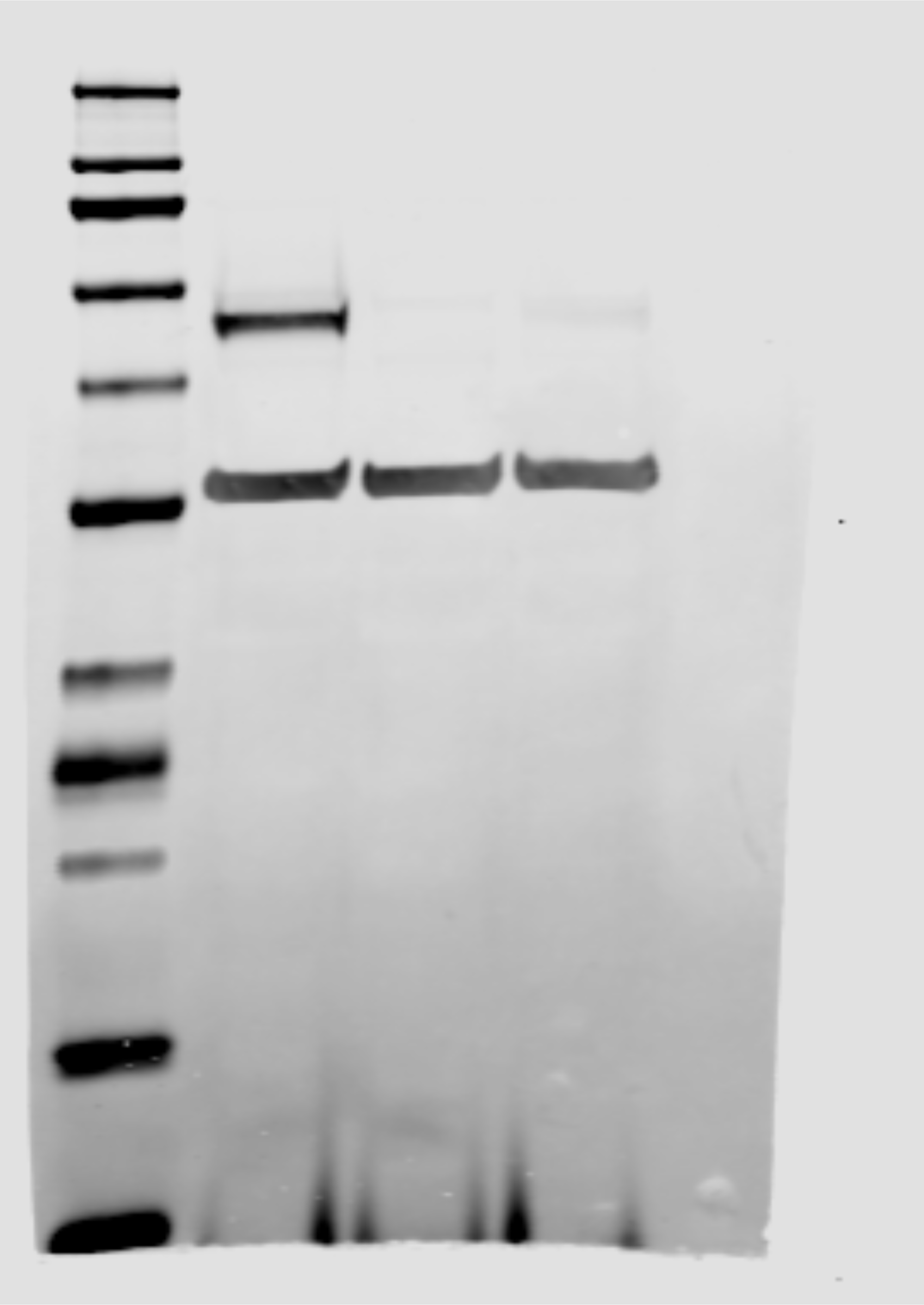

Supplement: Figure 5—source data 2. [file elife-111544-fig5-data2.zip › Figure 5_Source Data 2 - HT1080_WB_ACSL4.tif]

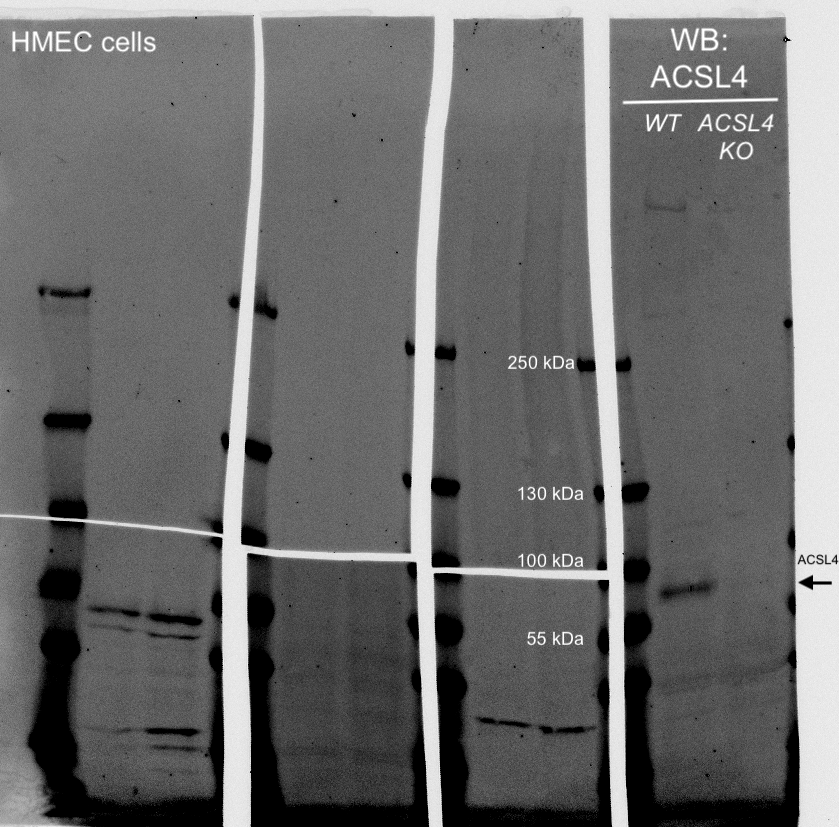

Supplement: Figure 5—figure supplement 1—source data 1. [file elife-111544-fig5-figsupp1-data1.zip › Figure 3-figure supplement 1-source data 1.TIF]

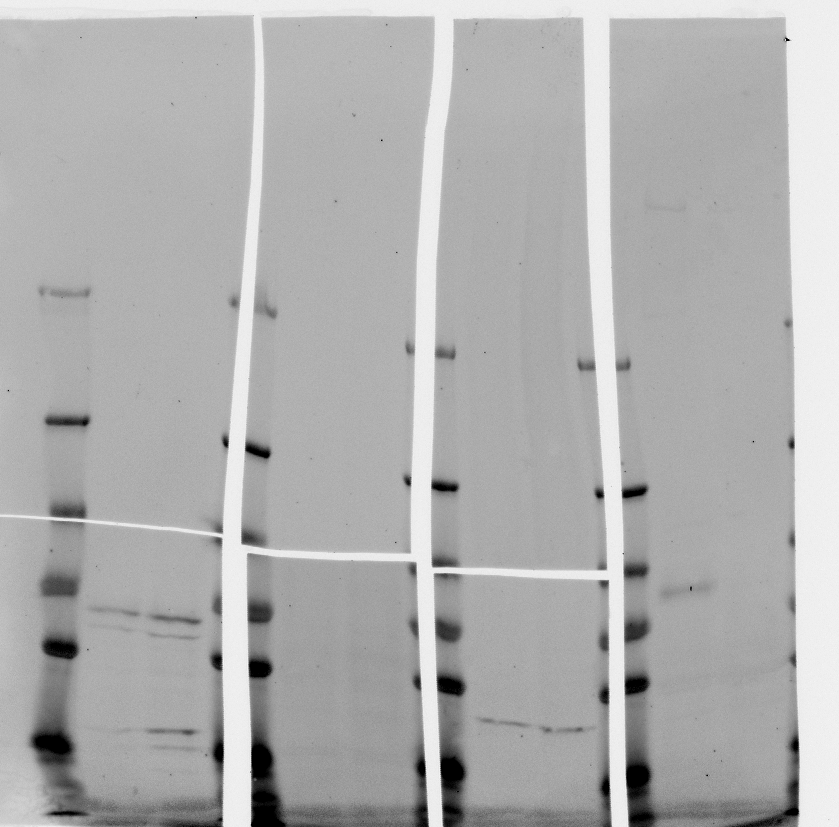

Supplement: Figure 5—figure supplement 1—source data 2. [file elife-111544-fig5-figsupp1-data2.zip › Figure 3-figure supplement 1-source data 2 - HMEC_WB_ACSL4.TIF]
