# Supplementary material for: Cell size modulates ferroptosis susceptibility
Source: eLife. 2026 Jun 10;15:RP111544. doi: 10.7554/eLife.111544 (PMC13252968; doi:10.7554/eLife.111544)
Supplement: Peer review history

# Editor report: eLife Assessment

**DOI:** 10.7554/eLife.111544.2.sa0

**Authors:** Alexis Barr

**Affiliations:** MRC Laboratory of Medical Sciences United Kingdom

**Keywords:** Convincing, Important

This **important** study highlights how cell size influences various cellular responses, with a particular focus on ferroptosis. The evidence presented is **convincing**, employing multiple model systems and experimental approaches to support the conclusions. This work will be of significant interest to the fields of cell size, ferroptosis, and cancer biology.

[Editors' note: this paper was reviewed by Review Commons.]

# Referee report: Reviewer #1 (Public review)

**DOI:** 10.7554/eLife.111544.2.sa1

Summary:

The study by Zatulovskiy et al. examined how cell size influences cell susceptibility to ferroptosis. The authors found a size dependence specifically for ferroptosis-inducing drug Era2, but not for other drugs. Using various human cell lines (HMEC, HT 1080, RPE 1), the authors generated populations of small and large G1 cells by FACS, CDK4/6 inhibition (palbociclib), or inducible cyclin D1 knockdown, and measured cell susceptibility to ferroptosis. Larger cells were more resistant than smaller cells. Mechanistically, larger cells showed reduced plasma membrane lipid peroxidation, higher glutathione concentrations, and changes in relevant cellular proteins levels, as analyzed using previously published data. Deleting ACSL4, which is involved in ferroptosis, partly eliminated the size dependence of ferroptosis. The work concludes that cell size is a key determinant of ferroptosis susceptibility. Overall, this work expands our understanding of how cell size is correlated with functional properties of cells, which can have implications for biomedical sciences.

Strengths:

The study establishes a credible link between cell size and susceptibility to ferroptosis, as induced by Era2. Experimental replication is sufficient, and key conclusions rely on data from multiple cell lines and on multiple approaches to manipulate cell size. This suggests that the conceptual findings made in this paper could reflect a more fundamental feature of mammalian cells. In addition, this work provides an interesting contrast to another recent study about size-dependency of ferroptosis (https://doi.org/10.1016/j.isci.2025.112363), where increased cell size heightened sensitivity to the GPX4 inhibitor RSL3.

Original Weaknesses:

Disentangling cell size effects from other confounding factors, such as the cell cycle or overall metabolic rate, is challenging, and the authors have managed to qualitatively prove that cell size influences Era2-induced ferroptosis. However, the quantitative nature of this link between cell size and susceptibility to ferroptosis remains somewhat unclear due to the confounding factors that are present in many of their experiments. Notably, the quantitative nature of this link could also be cell type and growth condition -dependent, which remain to be investigated in detail. It should also be noted that this work focused on cell culture studies, and it remains unclear how much the findings of this paper could influence therapeutic strategies in vivo.

Comments on revised version:

I would first like to emphasize that I find this work solid, and I think the authors have done good work with the revisions.

My only remaining recommendation is that the authors aim to more carefully examine the magnitude of the observed cell size-dependency in ferroptosis susceptibility. Their manuscript contains several experiments where the quantitative nature of this link remains unclear due to confounding factors, such as the cell cycle. For example, in Fig 2B&C, it seems that accumulation of cells in G1 (from ~60% to ~95%) decreases ferroptosis equally to the effect caused by cell volume doubling (from day 2 to day 4 of palbo treatment), suggesting that cell cycle has a much more pronounced effect on ferroptosis than cell size (especially when considering the size change from day 0 to day 2). However, the magnitude of the cell size effect is not consistent between all experiments shown. This is not surprising, as the authors use different approaches to changing cell size and different cell lines, but it makes the work more qualitative than quantitative. Notably, another confounding factor is the cell's metabolic/biosynthetic rate. It seems reasonable to assume that prolonged palbociclib treatment will decrease metabolic and protein synthesis rates (normalized to cell size), and this could make the cells less susceptible to ferroptosis. The rapamycin treatment results shown by the authors also support this notion. One approach to examining this could be to grow cells in various growth conditions to manipulate their growth & metabolic rate.

# Referee report: Reviewer #2 (Public review)

**DOI:** 10.7554/eLife.111544.2.sa2

Summary:

The authors set out to understand how cell phenotypes differ depending on the size of the cell, specifically here how cell size affects cell death. Using human cell lines (HMEC, HT-1080, RPE-1), the authors examined cell size through FACS sorting, CDK4/6 inhibition and inducible cyclin D1 knockdown. They identify that larger cells are more resistant to ferroptosis induced by system xc^-^ inhibition (erastin2), but more sensitive to GPX4 inhibition (RSL3), highlighting pathway-specific size dependencies.

Mechanistically, larger cells exhibited:

- Higher glutathione levels, supporting lipid peroxide detoxification

- Increased ferritin expression, promoting iron sequestration

- Lower ACSL4 levels, reducing incorporation of peroxidation-prone lipids

The findings are supported by high-throughput microscopy, flow cytometry (BODIPY-C11 lipid peroxidation assays), and proteomic analyses. The study concludes that cell size influences proteome composition and metabolic capacity, thereby shaping cell death decisions, an insight with implications for aging, cancer, and ferroptosis-based therapies.

Major Strengths:

- use of multiple cell lines to validate their findings

- use of multiple, complimentary approaches

- well designed screen and experiments throughout

- clearly written, logical flow and easy to follow

- relevance for multiple fields

Weaknesses:

- Lack of in-depth mechanistic investigation

- Experiments are all in vitro and so, as yet, it is uncertain what the in vivo consequence would be

General Assessment:

This study presents a mechanistic link between cell size and ferroptosis susceptibility. Using high-throughput microscopy, proteomics, and genetic perturbations across multiple human cell lines, the authors demonstrate that larger cells are more resistant to ferroptosis induced by system xc^-^ inhibition (erastin2). This resistance is attributed to elevated glutathione production, increased ferritin-mediated iron sequestration, and reduced ACSL4-dependent lipid peroxidation. The experimental design is rigorous and multifaceted, with consistent results across cell types and size manipulation methods. While the study is limited to in vitro systems, its conceptual and mechanistic insights lay the groundwork for future in vivo and translational investigations.

Advance:

This work is the first to systematically show that cell size directly influences ferroptosis susceptibility via proteome scaling. It reconciles previous findings that large cells are sensitized to GPX4 inhibition (RSL3) by demonstrating that the ferroptosis pathway targeted system xc^-^ vs GPX4 determines the direction of size-dependent vulnerability. The study provides a conceptual advance by positioning cell size as a regulatory axis in cell death decisions, and a mechanistic advance by identifying size-dependent changes in glutathione metabolism, ferritin levels, and ACSL4 expression.

Audience:

This research will be of interest to specialists in cell death, ferroptosis, redox biology, and cancer biology. It also holds relevance for aging researchers and translational scientists exploring ferroptosis-based therapies. The findings may influence how cell size heterogeneity is considered in therapeutic design, particularly in oncology and senescence-targeting strategies.

Comments on revised version:

We have no additional comments after revision. Thank you for addressing our initial queries.

# Referee report: Reviewer #3 (Public review)

**DOI:** 10.7554/eLife.111544.2.sa3

In this manuscript, Zatulovskiy and colleagues elaborate on their previous work describing cell size-dependent changes in the proteome by investigating whether these changes can be correlated in differences in cell physiology. Using a cleverly-designed high throughput screen, they searched for compounds that differently-sized cells display differential sensitivity towards. Their primary hit, Era2, is involved in the ferroptosis pathway and serves as the starting point for a detailed study of how excess cell size protects cells from ferroptosis-induced cell death via: (1) lower concentrations of ACSL4 (which produces peroxidation-prone PUFAs), (2) increased ferritin concentrations, and (3) increased GSH concentrations.

Overall, the experiments in this manuscript are well-designed and interpreted. It is an extremely well-written manuscript with a clear trajectory of logic.

Comments on the revised version:

The authors have addressed my original concerns adequately. I do not need to see it again, if there are further revisions.

# Author comment: Author response

**DOI:** 10.7554/eLife.111544.2.sa4

**Authors:** Evgeny Zatulovskiy, Magdalena B Murray, Shuyuan Zhang, Scott J Dixon, Jan M Skotheim

**Affiliations:** University of Cambridge Cambridge United Kingdom; Stanford University Stanford United States; Stanford University Stanford United States; Stanford University Stanford United States; Stanford University Stanford United States; Stanford University Stanford United States

**General Statements**

We thank all three reviewers for their professional and constructive feedback. We were pleased to see that all three reviewers support publication after revision. No one questions the premise that cell size influences ferroptosis susceptibility. The main concerns fall into two categories: (A) disentangling “Cell size vs cell cycle”, which is the biggest issue for Reviewer #1 and partially for #3. (B) Additional mechanistic tests including SLC7A11 and ferritin functional tests (Reviewer #2) and lysosomal iron (via LysoRhoNox) and some further ACSL4 experiments (Reviewer #3). Other reviewer concerns are more minor.

In our revision, we have addressed the reviewer’s specific criticisms with additional experiments as described below. We believe the constructive feedback from peer reviews helped us to significantly extend our mechanistic findings and strengthen the manuscript through revision.

**Point-by-point description of the revisions**

**Reviewer #1**:

Summary:

The study by Zatulovskiy et al. examined how cell size influences cell susceptibility to ferroptosis. The authors found a size dependence specifically for ferroptosis-inducing drug Era2, but not for other drugs. Using various human cell lines (HMEC, HT 1080, RPE 1), the authors generated populations of small and large G1 cells by FACS, CDK4/6 inhibition (palbociclib), or inducible cyclin D1 knockdown, and measured cell susceptibility to ferroptosis. Larger cells were more resistant than smaller cells. Mechanistically, larger cells showed reduced plasma membrane lipid peroxidation, higher glutathione concentrations, and changes in relevant cellular proteins levels, as analyzed using previously published data. Deleting ACSL4, which is involved in ferroptosis, partly eliminated the size dependence of ferroptosis. The work concludes that cell size is a key determinant of ferroptosis susceptibility.

My major concerns about this work focus on whether many of the results reflect cell size or cell cycle effects, and whether the FACS-based size-scaling analyses have some misleading features to their design & presentation. If these concerns can be addressed with new experiments, then the conclusions of this paper are justified. If these concerns cannot be addressed, then the authors should more directly acknowledge the alternative hypothesis that cell cycle effects may explain many of their results.

The experiments seem to be replicated sufficiently, and most conclusions rely on data from multiple cell lines. My minor comments focus on needs to provide statistics and method details, and on suggestions on how to improve text clarity, but these edits are easily done and don't require new experiments. Overall, this is an interesting study, and it should be published once the concerns below are addressed.

Major comments:

In experiments reported in Fig 1 and 2A, the authors sort small and large cells in G1, plate them, and later start the drug treatments & cell monitoring. Are these cells actively cycling (progressing in the cell cycle), and how fast? The large cells are likely to enter S phase earlier than the small cells, so by the time that the authors start their drug treatments, they may be comparing cells in different cell cycle stages, which could influence drug sensitivity more than cell size (as the authors also suggest later in Fig 2). This needs to be controlled for.

Furthermore, even if the cells remain in G1 after sorting until the drug treatments are started, the authors should address the fact that the drugs are present for a long time, thus targeting the cells in various cell cycle stages.

We agree with the reviewer that the cell cycle stage could affect ferroptosis susceptibility and could be a confounding effect in asynchronous cells. One of us (Dixon) reported the cell cycle effects on ferroptosis previously, and we observe them in this manuscript too (Fig. 2B,C,E). We now state this more clearly both in the Results and in the Discussion sections, where we write:

Line 159: “We note that non-arrested cells had a lower susceptibility to Era2-induced ferroptosis compared to cells that were arrested in G1 for 2-3 days, despite being smaller in size. This is likely due to the difference in the fraction of cells in different cell cycle phases between arrested and non-arrested conditions since cells in S/G2/M phases are known to be more resistant to ferroptosis than cells in G0/G1 phases (Rodencal et al, 2024; Kuganesan et al, 2023)”

Line 533: “Cells in G1 phase of the cell cycle were reported to be more susceptible to ferroptosis (Rodencal et al, 2024; Kuganesan et al, 2023), which suggested that ferroptosis inducers could be used in combination with cancer drugs, like the CDK4/6 inhibitor palbociclib, that arrest cells in G1 phase of the cell cycle (Herrera-Abreu et al, 2024). However, while CDK4/6 inhibitors arrest cells in G1, they do not inhibit cell growth, such that the longer they are arrested, the larger the cells grow (Lanz et al, 2022; Crozier et al, 2023; Manohar et al, 2023). This results in a complex, nonmonotonic ferroptotic response dynamics in cells treated with CDK4/6 inhibitors (Fig. 2B,E). Just following CDK4/6 inhibitor treatment, as more and more cells are arrested in G1 phase, cells become more sensitive to both RSL3- and erastin-induced ferroptosis (Kuganesan et al, 2023; Rodencal et al, 2024). However, the longer the cells are arrested, the larger they become, which further promotes their susceptibility to RSL3 (Fig. S1B) but reduces their susceptibility to Era2-induced ferroptosis (Fig. 2B). The fact that the cell cycle arrest and cell size increase have opposing effects on Era2-induced ferroptosis susceptibility could explain why different studies reported seemingly contradictory results, where sometimes an increased and sometimes a decreased or unchanged sensitivity to system x_c_^-^ inhibitors was observed depending on the cell type, duration and type of cell cycle arrest (Lee et al, 2024; Kuganesan et al, 2023; Rodencal et al, 2024). Such complex interplay between the cell cycle and cell size effects on ferroptosis suggests that combination therapies utilizing CDK4/6 inhibitors and ferroptosis inducers would have to carefully choose a dosage schedule.”

Given the potentially confounding effects of the cell cycle in cycling cells sorted by size, we performed an additional experiment, in which RPE-1 cells were pre-treated with the CDK4/6 inhibitor palbociclib to synchronize them in G1 phase prior to treatment. These cells were then continuously exposed to palbociclib during the Era2 treatment (Fig. 2C-E). RPE-1 cells pretreated with palbociclib for 2 and 4 days had the same cell cycle distribution with 94% of cells being arrested in G1, but with different sizes. Cells treated with palbociclib for 4 days were significantly larger and more resistant to Era2.

Additionally, in the experiment shown in Fig. 5E,F, where we FACS-sorted *WT* and *ACSL4 KO* HMEC cells by cell size, and then measured Era2 susceptibility, we pre-treated the cells with palbociclib for 24 h to synchronize them in G1 prior to the sorting. We then cultured the cells in the presence of palbociclib during the Era2 treatment to avoid the cell cycle effects observed in Fig. 2. In this case, we still observe that larger cells are more resistant to Era2, consistent with our conclusion that cell size protects against Era2-induced ferroptosis.

Can the G1 arrest-driven changes in drug susceptibility (Fig 2 C-D) be attributed to cell size? Can the authors rescue the palbociclib treatment with rapamycin or other growth inhibitors that allow size to remain small during G1 arrest?

We have attempted to perform these experiments, but when we co-treated the cells with palbociclib and mTORC inhibitors, we observed variable results, which are likely due to the fact that prolonged mTORC inhibition itself rewires cellular metabolism and reduces cell susceptibility to ferroptosis, as one of us (Dixon) found previously (Armenta et al. (2022), Ferroptosis inhibition by lysosome-dependent catabolism of extracellular protein. Cell Chemical Biology 29: 1588-1600.e7). Our results were consistent with this previous report and are now included in a new supporting figure panel (Fig. S3C):

Thus, upon palbociclib+rapamycin co-treatment there seems to be a competition between cell size-mediated and metabolism-mediated effects of mTORC inhibition on ferroptosis, which leads to variable outcomes.

In Fig 2E-F, is the cell cycle distribution of the samples influenced by CCND1 shRNA induction? Are the drug sensitivity effects due to cell size or cell cycle changes?

The CCND1 manipulation model is extensively characterized in our recent work cited in this manuscript (You et al. (2025), Cell size-dependent mRNA transcription drives proteome remodeling. 2025.10.30.685141 doi:10.1101/2025.10.30.685141). Indeed, CCND1 shRNA cells have a slightly elongated G1 phase due to a ~30% reduction in Cyclin D1 concentration: the G1 fraction changes from ~70% in wild-type to ~80% in CCND1 shRNA cells, which could potentially affect the ferroptosis susceptibility, but the additional results obtained on synchronized RPE-1 cells, described above (Fig. 2C-E), support the conclusion that the primary effect on Era2 sensitivity is due to cell size.

Can the authors address the meaningfulness of the FACS-based size-scaling results in cases where cell-to-cell variability is very large? For example, in Fig 4D&G, the results are so variable even in identically sized cells that the importance of the size-scaling pattern seems questionable.

We do observe variability in fluorescent probe-based measurements of GSH and lipid oxidation, which could be due to biological (natural cell heterogeneity) and/or technical (low sensitivity of the probes) reasons. However, when we look at binned data and compare the mean values ± s.e.m. for each bin, we observe a robust and reproducible trend (black line with dark-grey shaded area), even though the SD is quite broad (lighter shaded area). We believe such trends are meaningful when describing cell death in probabilistic terms as we do. I.e., the GSH measurement might not be precise enough to predict cell death for a given individual cell, but the statistical trend is clear and these measurements help predict cell death probabilities for cells of different sizes.

In Figs 4B-D, the cell size axis seems to have over 4-fold size variability, but when the authors show the analysis of this data (Figs 4E-G) the variability is only 2-fold. What was excluded and on what basis?

To address this point, we have now clarified in the Methods section how the data were processed and what data points we excluded from this analysis:

Line 671: “For all binned flow cytometry data plots, the cells below the second and above the 98th cell size percentiles were excluded to remove the extreme outliers. Then, the remaining data were binned by size and plotted as background-corrected average fluorescence intensity for each bin against the bin’s average cell size. Bins with fewer than 200 cells were excluded from the analysis to reduce noise.”

Typically, such pre-processing reduces the size range, mostly from the large-cell end, because of the long right tail of the size distribution containing a few very large cells.

Based on the methods section & figure legends of Fig 4B-I, the RPE cells were not pre-sorted to include only G1 cells, nor did the assay account for cell cycle differences. How can these data be used to explain results from earlier figures, where analyses were exclusively focused on size differences in G1?

This is a valid point: Cells in the GSH measurement experiment were not gated by Hoechst signal for G1 phase because the channel normally used for Hoechst staining was in this case occupied by the MCB probe. However, given the data in Fig. 4A,B showing that the GSH production machinery is superscaling when measured specifically in G1-phase cells, we believe the flow cytometry data in Fig. 4C-J showing GSH concentration increasing with cell size across the whole cell cycle is very likely true for G1 cells as well.

Minor comments:

I recommend clarifying in the early introduction that all size changes discussed are in the absence of DNA content increase.”

We have now clarified this in the introduction (Line 41 and Line 81).

The introduction seems to cite primary research and review paper in the same sentences, which is a bit misleading as the reviews don't seem to add new evidence.

We have removed review citations where they did not provide additional context.

OPTIONAL

In the second introduction paragraph, consider the classification/description of the three different mechanisms. Currently, it seems that these mechanisms are not independent of each other, and the details provided about each mechanism are inconsistent.”

We have now modified this paragraph to make the description more consistent.

Please provide statistics for the IC50 values reported based on Fig 1C. Were small and large cells statistically different? Are the IC50 values reported as +/- standard deviation or some other metric?

This has now been clarified in the text as follows:

“For example, at the 72 h time point, the Era2 IC50 was 28 ± 11 µM (mean ± SD) for large cells versus 2.0 ± 1.4 µM for small cells (Student’s t-test: p = 0.039) (Fig. 1C).”

Providing more insight into why Era2 and RSL3 treatments yield more opposite responses would be of great interest to the field.”

We agree this is an important point that should be discussed in more detail. In the field of ferroptosis, context-dependent (i.e., cell type-specific) effects are common and multiple groups including our own (Dixon) have published extensively on genes and mechanisms that can lead to differences between erastin2 and RSL3 sensitivity. For example, there are studies showing that the mTOR pathway or the p53 pathway can either prevent or promote ferroptosis, depending on the cell type and/or other currently unknown variables. To address more specifically the differences between Era2 and RSL3 in the context our observed cell-sizedependent response, we have now added more data and discussion. In the Results section we added panel 4B and the following text:

Line 359: “While the upregulation of GSH biosynthesis may promote the resistance of larger cells to ferroptosis, such an upregulation alone cannot explain why larger cells become more resistant to ferroptosis induced by the cystine import inhibitor Era2, but not, for example, by the GPX4 inhibitor RSL3 (Chan et al, 2025) (Figs. 2B, S1B). We found previously that upon mTORC1 inhibition cells can evade cystine deprivation-induced ferroptosis by uptake and catabolism of cysteine-rich extracellular proteins, mostly albumin (Armenta et al, 2022) (Fig. S3C). This process involves albumin degradation in lysosomes, predominantly by cathepsin B (CatB), and subsequent export of cystine from lysosomes to fuel the synthesis of glutathione. Large cells undergo proteome rearrangements similar to those occurring upon mTORC1 inhibition (Zatulovskiy et al, 2022). This suggests that large cells may upregulate CatB expression to bypass the Era2-induced cystine import inhibition via system xc-. To test this hypothesis, we used flow cytometry to measure how the expression of cathepsin B and the system xc- cystine/glutamate transporter SLC7A11 (xCT) scales with cell size (Fig. 4B). We found that SLC7A11 concentration modestly decreases, while CatB concentration significantly increases with cell size (Fig. 4B). This shift in the ratio between SLC7A11 and CatB supports the hypothesis that larger cells may rely less on cystine import via system xc- and thus become more resistant to system xc- inhibition by Era2.”

Additionally, in the Discussion we added the following:

Line 578: “We show that large cells may become resistant specifically to Era2 but not RSL3 through the upregulation of lysosomal function, particularly cathepsin B expression, which enables the uptake and catabolism of cysteine-rich extracellular proteins. A size-dependent shift in the ratio between SLC7A11 and cathepsin B makes large cells less dependent on cystine import via system xc-, and thus, more resistant to Era2. In addition to this, it was reported that RSL3 can induce ferroptosis independently of GPX4 and may target other selenoproteins (DeAngelo et al, 2025; Cheff et al, 2023), which could also contribute to the difference in sizedependent responses to RSL3 and Era2.”

Is the BODIPY-C11 labeling specific to plasma membrane, as suggested by the writing of the authors, or do the results shown integrate signals over all cell membranes?

We thank the reviewer for pointing this out. BODIPY-C11 581/591 stains many membranes in the cell, not just the plasma membrane. We have changed the wording in the manuscript to reflect this.

How exactly is gating done for the flow cytometry samples? Especially when analyzing size-scaling, the results are likely to be sensitive to outliers, such as those seen in Fig 4C (a subpopulation of very low CFSE stained cells). Can the authors clarify their methods and/or display supplementary figures with gating examples?

We have now specified our gating strategy in the Methods section (Line 663) and added a corresponding supplementary figure (Figure 4—figure supplement 1).

In Fig 4, total protein staining was used as a control, whereas Fig 5B b-actin was used as a control. Why did the authors rely on different controls approaches for essentially the same measurements? Are these controls comparable?

In our flow cytometry experiments, we consistently use live-cell total protein stain (CFSE) for live cells, and anti-Tubulin immunofluorescent staining for fixed cells, both of which scale in proportion to cell volume and act as a read-out for total cellular protein content (Lanz and Zatulovskiy et al., Mol Cell 2022; Berenson et al. MBoC 2019), which we use to calculate concentrations of other cellular components (analogous to loading controls). In Fig. 5B, betaActin is used as a reference - a protein whose concentration does not change with cell size, as opposed to ACSL4 whose concentration decreases with cell size. In this plot, both ACSL4 and beta-Actin amounts were normalized to alpha-Tubulin, which is analogous to a concentration calculation using loading control. This is now explained in more detail in the Figure legend.

**Reviewer #2**:

Zatulovskiy et al. demonstrate that cell size modulates susceptibility to ferroptosis, a form of iron-dependent cell death driven by lipid peroxidation. Using human cell lines (HMEC, HT-1080, RPE-1), the authors examined cell size through FACS sorting, CDK4/6 inhibition and inducible cyclin D1 knockdown. They found that larger cells are more resistant to ferroptosis induced by system xc^-^⁻ inhibition (erastin2), but more sensitive to GPX4 inhibition (RSL3), highlighting pathway-specific size dependencies.

Mechanistically, larger cells exhibited:

- Higher glutathione levels, supporting lipid peroxide detoxification

- Increased ferritin expression, promoting iron sequestration

- Lower ACSL4 levels, reducing incorporation of peroxidation-prone lipids

These findings were supported by high-throughput microscopy, flow cytometry (BODIPY-C11 lipid peroxidation assays), and proteomic analyses. The study concludes that cell size influences proteome composition and metabolic capacity, thereby shaping cell death decisions, an insight with implications for aging, cancer, and ferroptosis-based therapies.

Major Comments

(1) Direct evaluation of SLC7A11 abundance and function is needed

The opposite size-dependent effects of erastin2 and RSL3 strongly suggest a role for SLC7A11/system xc^-^ activity in size-dependent ferroptosis resistance. However, SLC7A11 levels were not quantified due to insufficient peptide detection in the proteomic data. o Direct measurement of SLC7A11 protein levels (immunoblotting or flow cytometry) in small vs large cells would test whether its expression scales with size.

a) Functional perturbation (siRNA/CRISPR knockdown) followed by erastin2 treatment would provide mechanistic validation. o Use of additional SLC7A11 inhibitors (e.g., sulfasalazine, sorafenib) could further test whether the size resistance phenotype is xc^-^-specific.

We agree that the difference in size-dependent responses to RSL3 and Era2 is an important point that needs further investigation and discussion, as other reviewers also pointed out. To address more specifically the differences between Era2 and RSL3 in the context of cell-sizedependent response, we have now added more data and discussion. In the Results section we added panel 4B measuring SLC7A11 and Cathepsin B scaling with cell size and the following text:

Line 359: “While the upregulation of GSH biosynthesis may promote the resistance of larger cells to ferroptosis, such an upregulation alone cannot explain why larger cells become more resistant to ferroptosis induced by the cystine import inhibitor Era2, but not, for example, by the GPX4 inhibitor RSL3 (Chan et al, 2025) (Figs. 2B, S1B). We found previously that upon mTORC1 inhibition cells can evade cystine deprivation-induced ferroptosis by uptake and catabolism of cysteine-rich extracellular proteins, mostly albumin (Armenta et al, 2022) (Fig. S3C). This process involves albumin degradation in lysosomes, predominantly by cathepsin B (CatB), and subsequent export of cystine from lysosomes to fuel the synthesis of glutathione. Large cells undergo proteome rearrangements similar to those occurring upon mTORC1 inhibition (Zatulovskiy et al, 2022). This suggests that large cells may upregulate CatB expression to bypass the Era2-induced cystine import inhibition via system xc-. To test this hypothesis, we used flow cytometry to measure how the expression of cathepsin B and the system xc- cystine/glutamate transporter SLC7A11 (xCT) scales with cell size (Fig. 4B). We found that SLC7A11 concentration modestly decreases, while CatB concentration significantly increases with cell size (Fig. 4B). This shift in the ratio between SLC7A11 and CatB supports the hypothesis that larger cells may rely less on cystine import via system xc- and thus become more resistant to system xc- inhibition by Era2.”

Additionally, in the Discussion we added the following:

Line 578: “We show that large cells may become resistant specifically to Era2 but not RSL3 through the upregulation of lysosomal function, particularly cathepsin B expression, which enables the uptake and catabolism of cysteine-rich extracellular proteins. A size-dependent shift in the ratio between SLC7A11 and cathepsin B makes large cells less dependent on cystine import via system xc^-^, and thus, more resistant to Era2. In addition to this, it was reported that RSL3 can induce ferroptosis independently of GPX4 and may target other selenoproteins (DeAngelo et al, 2025; Cheff et al, 2023), which could also contribute to the difference in sizedependent responses to RSL3 and Era2.”

(2) Functional tests of ferritin contribution to resistance are needed Although elevated ferritin (FTH1/FTL) levels in larger cells represent a strong correlational signal, definitive experimental evidence establishing causality is currently lacking. o Measuring the labile iron pool directly in size-stratified populations would strengthen the link. o Knockdown of FTH1 or FTL could reveal whether ferritin upregulation is necessary for the resistance of large cells to ferroptosis.

We thank the reviewer for raising this point. We have now completed additional experiments, as suggested by the reviewer, and found that iron chelation is unlikely to mediate the size-dependent response to Era2. We have modified the manuscript accordingly and added the following data and discussion to address this point:

Line 296: “The observed increase in ferritin concentration with cell size could therefore lead to additional Fe2+ ion chelation, which in turn would protect large cells from iron-dependent lipid peroxidation and ferroptosis. However, when we measured the concentration of labile intracellular Fe2+ using a fluorescent probe FerroOrange (Hirayama et al, 2020), we did not observe any size-dependent decrease in labile iron concentration (Fig. S2A). Previous work suggests a link between increased sequestration of ferrous iron in lysosomes and resistance to ferroptosis. It was reported that senescent cells, which are also large (Fig. S3A,B), gain resistance to ferroptosis through lysosomal alkalinization and sequestration of ferrous iron in lysosomes (Loo et al, 2025). We therefore tested whether the superscaling of lysosomes observed in large cells (Lanz et al, 2022; You et al, 2025) promotes Era2 resistance through lysosomal iron sequestration. To do this, we stained the cells with the lysosomal iron detection probe Lyso-FerroRed (Saimoto et al, 2025) and measured its scaling using flow cytometry (Fig. S2B). We observed that the amount of Lyso-FerroRed, and therefore, the amount of lysosomal iron, scaled in direct proportion to cell size, just like the total cellular protein content (Fig. S2B). These results indicate that iron chelation by ferritin and its sequestration in lysosomes are unlikely to play a crucial role in size-dependent decrease in Era2 sensitivity.”

(3) Relevance to senescence should be addressed experimentally or explicitly discussed

Given that senescent cells are enlarged and accumulate in aged and tumour tissues, testing senescent models for erastin2 resistance would greatly strengthen the physiological significance.”

We agree that an increase in cell size contributing to the resistance of senescent cells to ferroptosis is intriguing. We have now added a Supplementary Figure S3 and discussion of this point in the manuscript as follows:

Discussion line 552: “…our data suggest that previously reported resistance of senescent cells to ferroptosis can at least partially be due to the increased cell size, a well-established hallmark of senescence.”

Minor Comments

(1) Mechanistic nuance regarding RSL3 should be included

RSL3 has been reported to induce ferroptosis independently of GPX4 (PMID: 37087975, PMID: 40392234) and may target other selenoproteins such as TXNRD1. This nuance would help explain the observed divergence between RSL3 and erastin2 sensitivity across sizes.

We have now added this in the Discussion as suggested by the reviewer (line 583):

“In addition to this, it was reported that RSL3 can induce ferroptosis independently of GPX4 and may target other selenoproteins (DeAngelo et al, 2025; Cheff et al, 2023), which could also contribute to the difference in size-dependent responses to RSL3 and Era2.”

(2) Dynamic range of BODIPY-C11 assays needs commentary

Despite high erastin2 doses, the oxidized BODIPY signal remains close to DMSO levels. The authors should comment on whether this reflects high GSH buffering capacity, probe limitations, or other factors.”

We believe there are both technical (narrow dynamic range of the probe) and biological reasons for the relatively small (2-3 fold) difference in Oxidized-to-Non-oxidized BODIPY-C11 ratios between DMSO and Era2-treated cells. The biological reason is that the cells continue producing GSH until they fully deplete the cystine pool, which happens ~20-24 h after Era2 addition. Once the cystine pool is depleted, the cells very rapidly deplete GSH and initiate cell death. Therefore, there is only a short time window where cells are strongly depleted of GSH before dying. We see this small fraction of cells with a high Oxidized BODIPY-C11 signal in our flow cytometry experiments and in previous microscopy analysis of BODIPY-C11 (Murray et al., Protocol for detection of ferroptosis in cultured cells. STAR Protoc. 2023), but at our chosen time point (20h Era2) most cells are not as bright because we aimed to analyze the population before the onset of widespread cell death.

(3) Western blot for shCycD1 depletion should be included

CycD1 depletion usually causes cells to stop proliferating, which is not the case here. Therefore, depletion must be partial. The level of depletion should be shown by immunblotting.”

The CCND1 manipulation model is extensively characterized in our recent work cited in this manuscript (You et al. (2025), Cell size-dependent mRNA transcription drives proteome remodeling. 2025.10.30.685141 doi:10.1101/2025.10.30.685141). CCND1 shRNA cells do not fully arrest in G0/G1 because the concentration of Cyclin D1 protein in this system is only partially decreased, as the reviewer noted. As a result, the cells have a slightly elongated G1 phase due to a ~30% reduction in Cyclin D1 concentration, but continue to proliferate. The G1 fraction changes from ~70% in wild-type to ~80% in CCND1 shRNA cells.

**Reviewer #2 (Significance):**

General Assessment: This study presents a mechanistic link between cell size and ferroptosis susceptibility. Using high-throughput microscopy, proteomics, and genetic perturbations across multiple human cell lines, the authors demonstrate that larger cells are more resistant to ferroptosis induced by system xc^-^ inhibition (erastin2). This resistance is attributed to elevated glutathione production, increased ferritinmediated iron sequestration, and reduced ACSL4-dependent lipid peroxidation. The experimental design is rigorous and multifaceted, with consistent results across cell types and size manipulation methods. While the study is limited to in vitro systems, its conceptual and mechanistic insights lay the groundwork for future in vivo and translational investigations.

Advance: This work is the first to systematically show that cell size directly influences ferroptosis susceptibility via proteome scaling. It reconciles previous findings that large cells are sensitized to GPX4 inhibition (RSL3) by demonstrating that the ferroptosis pathway targeted system xc^-^ vs GPX4 determines the direction of size-dependent vulnerability. The study provides a conceptual advance by positioning cell size as a regulatory axis in cell death decisions, and a mechanistic advance by identifying size-dependent changes in glutathione metabolism, ferritin levels, and ACSL4 expression.

Audience: This research will be of interest to specialists in cell death, ferroptosis, redox biology, and cancer biology. It also holds relevance for aging researchers and translational scientists exploring ferroptosis-based therapies. The findings may influence how cell size heterogeneity is considered in therapeutic design, particularly in oncology and senescence-targeting strategies.

Field of Expertise: Translational cancer biology, cell cycle regulation, proteomics, therapy resistance, molecular mechanisms of cell death.

We thank Reviewer #2 for their careful and constructive assessment of our manuscript. We were happy that they appreciated the rigor of our multifaceted approach. We are also grateful for their thoughtful perspective on the conceptual and mechanistic advances, and for highlighting the broader relevance of this work to ferroptosis biology, redox regulation, cancer and aging research.

**Reviewer #3 (Evidence, reproducibility and clarity):**

In this manuscript, Zatulovskiy and colleagues elaborate on their previous work describing cell size-dependent changes in the proteome by investigating whether these changes can be correlated in differences in cell physiology. Using a cleverly-designed high throughput screen, they searched for compounds that differently-sized cells display differential sensitivity towards. Their primary hit, Era2, is involved in the ferroptosis pathway and serves as the starting point for a detailed study of how excess cell size protects cells from ferroptosis-induced cell death via: (1) lower concentrations of ACSL4 (which produces peroxidation-prone PUFAs), (2) increased ferritin concentrations, and (3) increased GSH concentrations.

Overall, the experiments in this manuscript are well-designed and interpreted. It is an extremely well-written manuscript with a clear trajectory of logic. I have only a few major concerns that should be addressed before publication:

We thank Reviewer #3 for their careful reading of the manuscript and for the clear summary of our study and its central findings. We appreciate their positive assessment of the experimental design, interpretation, and overall clarity of the writing and logical flow. We are also grateful for their constructive feedback and take their major concerns seriously; we have addressed each point in detail below.

Major concerns:

(1) In Figure 3E, the authors gate their flow cytometry data using SYTOX so that they are only analyzing live cells. Based on their gating scheme, it seems like there are really a lot of dead cells. Presumably the cells that died were the most sensitive to Era2, so it seems an oversight to discard these cells. Of course, it is not appropriate to analyze dead cells, but this could potentially be solved by using a shorter treatment duration than 24 hours wherein fewer cells die.”

This is a good point. To address it, we have now replaced this panel with a time point where most cells are still alive (20 h, 0.2 µM Era2), as suggested by the reviewer (Fig. 3E,F). This did not change the conclusion that BODIPY-C11 oxidation decreases with cell size.

(2) In Figure 5, are the small, medium, and large bins for ACSL4 KO cells the same as for WT cells? If the ACSL4 KO cells are just bigger to begin with, this could explain why the "small" bin has greater cell survival than the WT small bin. Moreover, is the overlap between the three bins the same in the WT and KO cells?

This is an important point that we now address with data shown in Fig. S4B. We have now added a Supplementary Figure S4B to show the relative size of small, medium, and large *WT* and *ACSL4 KO* HMEC cells. As seen from this graph, the *ACSL4 KO* cells are not bigger than *WT* cells. Importantly, the fold-range between the small and large FACS-sorted cells is similar (~1.9 to 2-fold).

(3) Loo, et al. Nat Comms 2025 similarly found that senescent cells (which are enlarged) are resistant to ferroptosis using the same inhibitor as the authors. In contrast to the authors, they show that this is due to lysosomal alkalinization and sequestration of ferrous iron in lysosomes. Given that Lanz et al. 2022 found that lysosomal components super-scale with cell size, it seems like this would be an important hypothesis to address. Free lysosomal iron can be easily measured with the LysoRhoNox stain. Loo et al. was able to restore ferroptosis sensitivity in senescent cells using the V-ATPase activator EN6, so it would be important for the authors to address whether this (or similar) treatment would have the same effect in enlarged cells.

This is an excellent point. We have now performed this experiment and added it to the manuscript, as suggested by the reviewer. Based on the Lyso-FerroRed staining (another brand name for the LysoRhoNox probe), we do not see an increase in lysosomal iron sequestration in large cells (Fig. S2B):

Line 301: “Previous work suggests a link between increased sequestration of ferrous iron in lysosomes and resistance to ferroptosis. It was reported that senescent cells, which are also large (Fig. S3A,B), gain resistance to ferroptosis through lysosomal alkalinization and sequestration of ferrous iron in lysosomes (Loo et al, 2025). We therefore tested whether the superscaling of lysosomes observed in large cells (Lanz et al, 2022; You et al, 2025) promotes Era2 resistance through lysosomal iron sequestration. To do this, we stained the cells with the lysosomal iron detection probe Lyso-FerroRed (Saimoto et al, 2025) and measured its scaling using flow cytometry (Fig. S2B). We observed that the amount of Lyso-FerroRed, and therefore, the amount of lysosomal iron, scaled in direct proportion to cell size, just like the total cellular protein content (Fig. S2B). These results indicate that iron chelation by ferritin and its sequestration in lysosomes are unlikely to play a crucial role in size-dependent decrease in Era2 sensitivity.”

Minor concerns:

(1) It would be helpful if this manuscript were re-submitted with line numbers to more easily reference the text.

We have added line numbers for convenience.

(2) In Figure 5A and other figures that reproduce data from Lanz et al. 2022, it would be helpful to have a summary curve for the overall abundance of each protein rather than only the individual peptide curves. These plots (particularly Figure 5A) are difficult to interpret since some peptides were presumably more abundant / measured with higher confidence than others.

We have added the average ACSL4 protein slope line to Fig. 5A.

(3) In Figure 5, the authors show the validation of the ACSL4 KO HT-1080 cell line but not HMEC, even though both are used in this figure. It would be useful to show both. Additionally, the authors switch back and forth between the two cell lines for this figure, and it is not clear why.

We have added the HMEC *ACSL4 KO* validation Western blot in Fig. S4A.

For the BODIPY oxidation experiment (Fig. 5D), we used HT-1080 instead of HMEC because HT1080 cells are sensitive to lower concentrations of Era2, and therefore, we could better optimize the Era2 concentrations and treatment durations to measure BODIPY oxidation at the time point when most cells are still alive but demonstrate a pronounced oxidized BODIPY signal.

(4) In Figure 5B, the authors use antibody-based staining of ACSL4 and flow cytometry to correlate a loss of ACSL4 expression with increased cell size, validating the proteomics data in Figure 5A. This does not seem like a good way to do this. Firstly, fixing cells with formaldehyde alters their size (is this proportional across differently sized cells? It's impossible to know), which makes it inappropriate to use SSC as a proxy for size in this particular situation. Secondly, the normalization scheme here doesn't make sense. If actin was used as a reference protein, why was tubulin used to normalize ACSL4 abundance? Overall, this seems like a very round-about experiment that could have just been addressed by doing a simple western blot with the four size bins sorted from live cells (as it was in the proteomics). If the issue is that ACSL4 is not detectable by western in the HMEC cells, another solution would be plating the live, sorted bins on coverslips and measuring by IF (or using the HT-1080 cells).

We prefer IF flow cytometry to Western blotting for protein scaling analysis because it is more quantitative and provides cell size and protein content information for each individual cell. While in principle, different-sized cells might change their size differently during fixation, the cells that were larger or smaller prior to the fixation remain larger or smaller after fixation as well.

Therefore, the SSC measurement after fixation still provides reliable information on size ranking, even if SSC does not perfectly linearly scale with cell volume. We do not use the SSC information to calculate protein concentrations here. Instead, we divide the amount of our protein of interest in the cell by the amount of constitutively-expressed Tubulin, which acts as an analogue of a loading control in this experiment. In Fig. 5B, both ACSL4 and Actin were normalized to Tubulin to estimate their concentrations. Actin is used just as a reference protein to show how the concentration of a perfectly scaling protein remains constant across cell size, as opposed to the sub-scaling ACSL4. Tubulin in this case was used as a proxy for total cellular protein content, which scales linearly in proportion to cell volume. This approach for determining the scaling behaviors of different proteins was previously validated in Lanz et al., Mol Cell 2022.

(5) In Figure 5E/5F, the authors pre-arrest the cells in G1 with palbociclib before size-sorting them. The pre-arrest is not done in other experiments using this cell line for sizesorting, so it would be important for the authors to comment on why this was done for this experiment but not others.”

As we found in Fig. 2B-E, the cell cycle has confounding effects on size-dependent ferroptosis susceptibility measurements (as discussed in detail in our response to the first major point of Reviewer #1 above). Briefly, to avoid these confounding effects and isolate the effects of cell size from the effects of the cell cycle, we pre-synchronized the cells with 24 h treatment with palbociclib in Fig. 5E,F. This is now better clarified in the text, as follows:

Line 456: “In this experiment, we synchronized cells in G1 phase using palbociclib prior to cell sorting and also incubated the sorted cells in the presence of palbociclib during Era2 treatment to isolate cell size effects from the previously observed confounding effects of the cell cycle on ferroptosis (Fig. 2B,E).”

(6) Conceptually, it is difficult for me to understand why large cell size sensitizes cells to GPX4 inhibition but confers resistance to Era2 treatment. Particularly given the pathway described in Figure 3A, I am having trouble understanding why these would convey such opposing phenotypes. Shouldn't the extra ferritin in the bigger cells also help them cope with GPX4 inhibition if, as the authors state in the discussion, the increased sensitivity to the GPX4 inhibitor is reported to be mediated by (among other things) iron accumulation? A deeper discussion of this seeming-incongruity would be helpful for contextualizing the broader role of cell size in determining ferroptosis sensitivity.

We agree this is an important point, which was also raised by the other reviewers. As such, we note that context-dependent (i.e., cell type-specific) effects are common in the ferroptosis field, and multiple groups including our own (Dixon) have published extensively on genes and mechanisms that can lead to differences between erastin2 and RSL3. For example, there are studies showing that the mTOR pathway or the p53 pathway can both prevent and promote ferroptosis, depending on the cell type or some other hidden variable.

To better address the differences between Era2 and RSL3 in the context of the cell-sizedependent response, we have now added more data and discussion. In the Results section we added panel 4B and the following text:

Line 359: “While the upregulation of GSH biosynthesis may promote the resistance of larger cells to ferroptosis, such an upregulation alone cannot explain why larger cells become more resistant to ferroptosis induced by the cystine import inhibitor Era2, but not, for example, by the GPX4 inhibitor RSL3 (Chan et al, 2025) (Figs. 2B, S1B). We found previously that upon mTORC1 inhibition cells can evade cystine deprivation-induced ferroptosis by uptake and catabolism of cysteine-rich extracellular proteins, mostly albumin (Armenta et al, 2022) (Fig. S3C). This process involves albumin degradation in lysosomes, predominantly by cathepsin B (CatB), and subsequent export of cystine from lysosomes to fuel the synthesis of glutathione. Large cells undergo proteome rearrangements similar to those occurring upon mTORC1 inhibition (Zatulovskiy et al, 2022). This suggests that large cells may upregulate CatB expression to bypass the Era2-induced cystine import inhibition via system xc-. To test this hypothesis, we used flow cytometry to measure how the expression of cathepsin B and the system xc- cystine/glutamate transporter SLC7A11 (xCT) scales with cell size (Fig. 4B). We found that SLC7A11 concentration modestly decreases, while CatB concentration significantly increases with cell size (Fig. 4B). This shift in the ratio between SLC7A11 and CatB supports the hypothesis that larger cells may rely less on cystine import via system xc- and thus become more resistant to system xc- inhibition by Era2.”

Additionally, in the Discussion we added the following:

Line 578: “We show that large cells may become resistant specifically to Era2 but not RSL3 through the upregulation of lysosomal function, particularly cathepsin B expression, which enables the uptake and catabolism of cysteine-rich extracellular proteins. A size-dependent shift in the ratio between SLC7A11 and cathepsin B makes large cells less dependent on cystine import via system xc-, and thus, more resistant to Era2. In addition to this, it was reported that RSL3 can induce ferroptosis independently of GPX4 and may target other selenoproteins (DeAngelo et al, 2025; Cheff et al, 2023), which could also contribute to the difference in sizedependent responses to RSL3 and Era2.”
